# Carboxybetaine-modified succinylated chitosan-based beads encourage pancreatic β-cells (Min-6) to form islet-like spheroids under in vitro conditions

**DOI:** 10.1007/s10856-017-6018-0

**Published:** 2017-12-30

**Authors:** Valeria Perugini, Mark Best, Sandeep Kumar, Anna L. Guildford, Adrian J. Bone, Wendy M. Macfarlane, Matteo Santin, Gary J. Phillips

**Affiliations:** 1 School of Pharmacy and Biomolecular Sciences, University of Brighton, Huxley Building Lewes Road, Brighton, BN2 4GJ UK; 2 Cellon S.A., ZAE Robert Steichen, 16 rue Hèierchen, L-4940 Bascharage, Luxembourg

## Abstract

In vitro, pancreatic β-cells tend to reduce their ability to aggregate into islets and lose insulin-producing ability, likely due to insufficient cell–cell and cell–matrix interactions that are essential for β-cell retention, viability and functionality. In response to these needs, surfaces of succinylated chitosan-based beads (NSC) were modified with zwitterionic carboxy-betaine (CB) moieties, a compatible osmolyte known to regulate cellular hydration state, and used to promote the formation of β-cell spheroids using a conventional 2D cell culture technique. The NSC were synthesised by ionic gelation and surface-functionalised with CB using carbodiimide chemistry. Scanning electron microscopy (SEM), dynamic laser scattering (DLS) and Fourier transform infrared spectroscopy (FTIR) were employed as characterisation tools to confirm the successful modification of the succinylated chitosan material into spherical beads with rough surfaces and a diameter of 0.4 µm. NSC with and without CB were re-suspended at concentrations of 0.1, 0.3 and 0.6 mg/mL in saline medium and tested in vitro with MIN6 murine pancreatic β-cell line. Results showed that a concentration of 0.3 mg/mL, NSC-CB encouraged pancreatic MIN6 cells to proliferate and form spheroids via E-cadherin and Pdx-1 activation within 48 h in culture. These spheroids, with a size of approximately 80 µm, exhibited high cell viability and enhanced insulin protein expression and secretion when compared to cells organised by the non-modified beads.

## Introduction

Pancreatic islets, also known as Langerhans islets, are spherical units that are comprised of clusters of cells distributed throughout the pancreas [1]. The β-cells are one of the major cell types within islets and are involved in storing and releasing insulin, a hormone that is critical in the regulation of blood glucose levels [2]. β-cell activities are tightly controlled by neighbouring cells and the extracellular matrix (ECM) that closely interact with β-cells through cell surface proteins (e.g. E-cadherin) and gap-junctions [3]. Direct contacts between cell–cell and cell–matrix are therefore essential to maintain the survival and function of β-cells [4]. During in vitro cell culture, β-cells are isolated from their native tissues and grow on traditional tissue culture plates coated with non-adhesive substances (e.g. agarose) or roller flasks and shakers. These current techniques have been shown to disrupt both cell–cell and cell–matrix interactions by inducing changes in gene expression and β-cell phenotype. Progress in the development of three dimensional (3D) culture methods has addressed these limitations [5] through the use of biocompatible materials for microencapsulation or layer-by-layer coating of single islets [6] that are capable of mimicking the natural cellular microenvironment and enhancing β-cell activities [7]. For example, pancreatic MIN6 cells demonstrated a better survival rate and glucose responsiveness to insulin over a ten day incubation when they were encapsulated in cell adhesive peptide (RGD)-modifed PEG hydrogels [8]. However, even the most advanced in vitro 3D culture approaches lack important features needed to reconstitute the in vivo β-cell microenvironment [9, 10]. Particle-based materials, especially beads, have attracted some interest for many technological applications and shown varying degrees of success as culture systems [11]. These materials offer advantages such as high tissue permeability [12], lower enzymatic degradation [13] and large surface area [14]. To date, beads have commonly been prepared using natural polymers, such as chitosan, a polysaccharide that possesses excellent biodegradable, bioadhesive and biocompatible properties [15].

Chitosan is a naturally occurring biopolymer produced on an industrial scale for use in the pharmaceutical, cosmetics, agriculture and food sectors [16]. It is derived from the deacetylation of chitin, a major by-product of the marine and fishery industry, to different degrees by reaction with strong alkali. Deacetylation of chitin forms β-(1→4)-linked 2-amino-2-deoxy-D-glucopyranose (GlcN, D-unit) and 2-acetamido-2-deoxy-D-glucopyranose (GlcAc, A-unit) units in chitosan, the ratio of which can be measured using NMR to yield the degree of deactylation as a percentage (% DD). The removal of acetyl groups results in the presence of free amino functionalities in chitosan, and is responsible for its polycationic nature in acidic solutions [17]. However, the poor solubility of unmodified chitosan in both water and organic solvents has strictly limited its final application [18]. This limitation has been overcome using N-succinyl-chitosan (SNC) an acyl derivate of chitosan that is prepared by introducing succinyl groups onto the N-terminals of the chitosan glucosamine units [19]. As such, NSC presents favourable properties such as good biocompatibility and low toxicity, but it still faces problems with regards to biomolecular recognition.

Recently, aqueous solutions of carboxy-betaine (CB) derivatives, which are known to be zwitterionic materials, have drawn special attention due to their anti-biofouling properties of resisting protein adsorption and biofilm formation on a variety of substrates and surfaces as well as providing the capability for further biomaterial functionalisation [20]. Also conferring protection to the cells against environmental stresses like osmotic irregularity, adverse temperatures, and dehydration [21], CB is considered a promising therapeutic agent in the treatment of a number of diseases including Alzheimer [22], hepatopathy [23] and cancer [24]. The overall hydration property of CB is probably related to its capacity to form hydrogen-bonded interactions with a range of organic and inorganic acids owing to a high electron density on its two carboxylic oxygen atoms. This is responsible for both the structure and properties of CB and has been shown to greatly support cell adhesion and proliferation in several types of cells [25]. In this study, betaine-modified NSC-beads (NSC-CB) were considered to have the potential to act as a simple innovative in vitro cultivation system for the aggregation and formation of MIN6 cell spheroids without the use of complex equipment including rotary shaker, hanging-drop plate and artificial matrix. Therefore, NSC-based beads have been created and assessed for their potential to (i) be easily modified with CB molecules; (ii) act as a scaffold system to encourage formation of pancreatic β-cell (MIN-6) spheroids under 2D culture conditions and (iii) control metabolic functionality including the storage and release of insulin by the MIN6 cells via E-cadherin and Pdx-1.

## Materials and methods

### Synthesis and functionalisation of N-succinylated chitosan beads

Chitosan (Aldrich-Sigma, UK) was dissolved in acetic acid and diluted 1:1 with methanol before derivatisation with 4% (w/v) succinic anhydride in acetone solution (SC) (Fischer Scientific, UK). Ionic gelation at a 4:1 volume ratio with 1 mg/mL sodium tripolyphosphate (NaTPP) (Aldrich-Sigma, UK) in deionised H_2_O was used to form *N*-succinylated chitosan beads (NSC). The surface was tethered with betaine (CB) (Aldrich-Sigma, UK) by previous activation of the relevant functional groups with 10× molar excess N-(3-dimethylaminopropyl)-N′-ethylcarbodiimide hydro-chloride (EDC) and a 25× molar excess N-hydroxysuccinimide (NHS) in 0.1 M MES buffer pH 6.0 (Aldrich-Sigma, UK). NSC-CB were desalted into deionised H_2_O using dialysis with 3.5 kDa MW cut off cellulose tubing and filtered through a 0.45µm-pore syringe before lyophilisation.

### Characterisation analysis of NSC with and without CB

Fourier transform infrared spectroscopy (FTIR) was carried out using a Perkin Elmer Spectrum 65 and used to confirm both the N-succinylation of the chitosan and its functionalisation with CB; whereas both morphology and structure of NSC and NSC-CB were investigated using a Zeiss Σigma™ field emission gun scanning transmission electron microscope (SEM, FEG-STEM). The size of the beads was evaluated using a dynamic light scattering system (DLS) from Zetasizer ZS90 (Malvern Instruments Ltd., UK).

### Cell culture and seeding

The mouse pancreatic β MIN6 cells were maintained in low-glucose Dulbecco’s modified Eagles’s medium (DMEM, Gibco^®^ Life Technologies Ltd., UK) supplemented with 10% v/v foetal bovine serum (FBS, Aldrich-Sigma, UK) in an incubator at 37 °C and 5% CO_2_ for 1 complete passage before use in experiments.

MIN6 cells (passage 32) were seeded at an optimal density of 4 × 10^4^/mL [26] and cultured with both NSC and NSC-CB at concentrations of 0.1, 0.3, and 0.6 mg/mL according to a patented method [27] for 48 h incubation.

Cells seeded on tissue culture grade polystyrene plates (TCP) were used as a traditional 2D negative control; while a positive control of 3-D spheroid formation was achieved by seeding MIN6 cells on commercial Matrigel substrates (BDBioscience, UK), known to induce cell aggregation [28].

### Morphology and morphometric analysis of MIN6 cells

MIN6 cells treated with NSC and NSC-CB were imaged using phase contrast microscopy (Leica DM2500) with an ×10 magnification connected to a digital camera (Canone LM Scope) within 48 h incubation. The size of each spheroid was determined using ImageJ software (ImageJ, 1.42q, National Institutes of Health, NHI) and expressed as mean diameter ± standard deviation (*n* = 12).

### Cell viability and toxicity

To identify both live and dead cells, MIN6 cells were stained with 50 µl of Hoesch-Propidium Iodide (HPI, Sigma Aldrich, UK) (ratio 1:1) and imaged using a 10× objective lens attached to an epi-fluorescence Zeiss Axiovert 25 UV microscope 48 h after plating. Fluorescence of the tested cells was quantified using an established protocol [29] while data were expressed as percentage of either viable and apoptotic cells (*n* = 12).

The supernatants from each sample were collected and analysed using a cytotoxicity assay kit based on LDH release (Promega, UK). Briefly, a 1:1 ratio of sample was mixed with LDH substrate (provided in the kit), and incubated for 30 min at room temperature protected from light. The enzyme reaction was then stopped by adding an equal volume of 1 M acetic acid, with absorbance measured in triplicate at 492 nm with spectrophotometer (ThermoAskin Accent, UK).

### Immunofluorescent staining of MIN6 cells

MIN6 cells were fixed in chilled methanol for 15 min at 20 °C, rinsed with phosphate-buffered saline (PBS), and blocked with bovine serum albumin (BSA; Sigma Aldrich, UK) to prevent nonspecific staining. The cells were incubated in a polyclonal insulin primary antibody (1:75, Cell Signalling, UK) and a mouse monoclonal antibody against E-cadherin (1:100; AbCam, UK) overnight at 4 °C. They were then rinsed in PBS and immunolabelled with secondary antibodies (Alexa Fluor 488 anti-rabbit IgG and Alexa Fluor 488 anti-rat IgG, 10 μg/mL; Invitrogen, UK) in the dark at room temperature for 1 h. The stained cells were washed again, incubated in 11 μM 4′-6-diamidino-2-phenylindole (DAPI, Invitrogen, UK) to counterstain the nuclei and imaged using a Leica TCS SP5 laser scanning confocal microscope with an objective lens ×20. E-cadherin regulation was then evaluated using a feature of ImageJ, Regions of Interests (ROI) Manager (http://mirror.imagej.net), and expressed as means ± standard deviation of fluorescent E-cadherin expressing cells (*n* = 12).

### Insulin secretion

Insulin concentration in the supernatants collected from the cell culture study was assessed using an anti-mouse insulin ELISA kit (Millipore, UK). The method followed manufacturer instructions as stated for cell supernatant samples. The absorbance was read at 450 nm by a spectrophotometer plate reader (Biotek ELx800, UK) at 450 nm (*n* = 6); whereas insulin values were normalised against the total protein content (Bradford assay, Bio-rad, UK) and expressed as ng/mg of protein for each sample (*n* = 6).

### Western blotting

MIN6 cells were detached with 0.02 % EDTA and prepared for protein extraction using a solution of lysis RIPA buffer supplemented with 50 µg/mL protease inhibitors (Aldrich-Sigma, UK). The total protein content was then measured by the Bradford assay while their equivalent amounts (30 µg) were separated by sodium dodecyl sulfate (SDS)-gel electrophoresis on 10% polyacrylamide gel (Biorad, UK) and blotted against a nitrocellulose membrane (Amersham, UK) at a constant current of 20 mA overnight. Membranes were blocked with 0.01% w/v BSA in a Tris-buffered saline (TBS)-Tween solution (TBST, 0.5 M Trizma, 1.5 M NaCl and 0.2 % Tween-20, pH 7.4) and then incubated with specific primary antibodies including anti-Ki67, and anti-Pdx-1 (1:100; Abcam, UK) overnight at 4 °C. After being washed with TBST three times, membranes were treated with appropriate HRP-conjugated secondary antibodies (1:1000; Invitrogen, UK) for 1 h at room temperature and developed using an ECL detection kit according to the manufacturer’s instructions (Amersham, UK). GAPDH was used as a positive control.

### Statistical analysis

Statistical analysis was carried out using ANOVA test while quantitative data were expressed as mean ± standard deviation. A level of significance of *p* ≤ 0.05 was regarded as statistically significant.

## Results

### Characterisation and analysis of NSC with and without CB

**Fig. 1:**
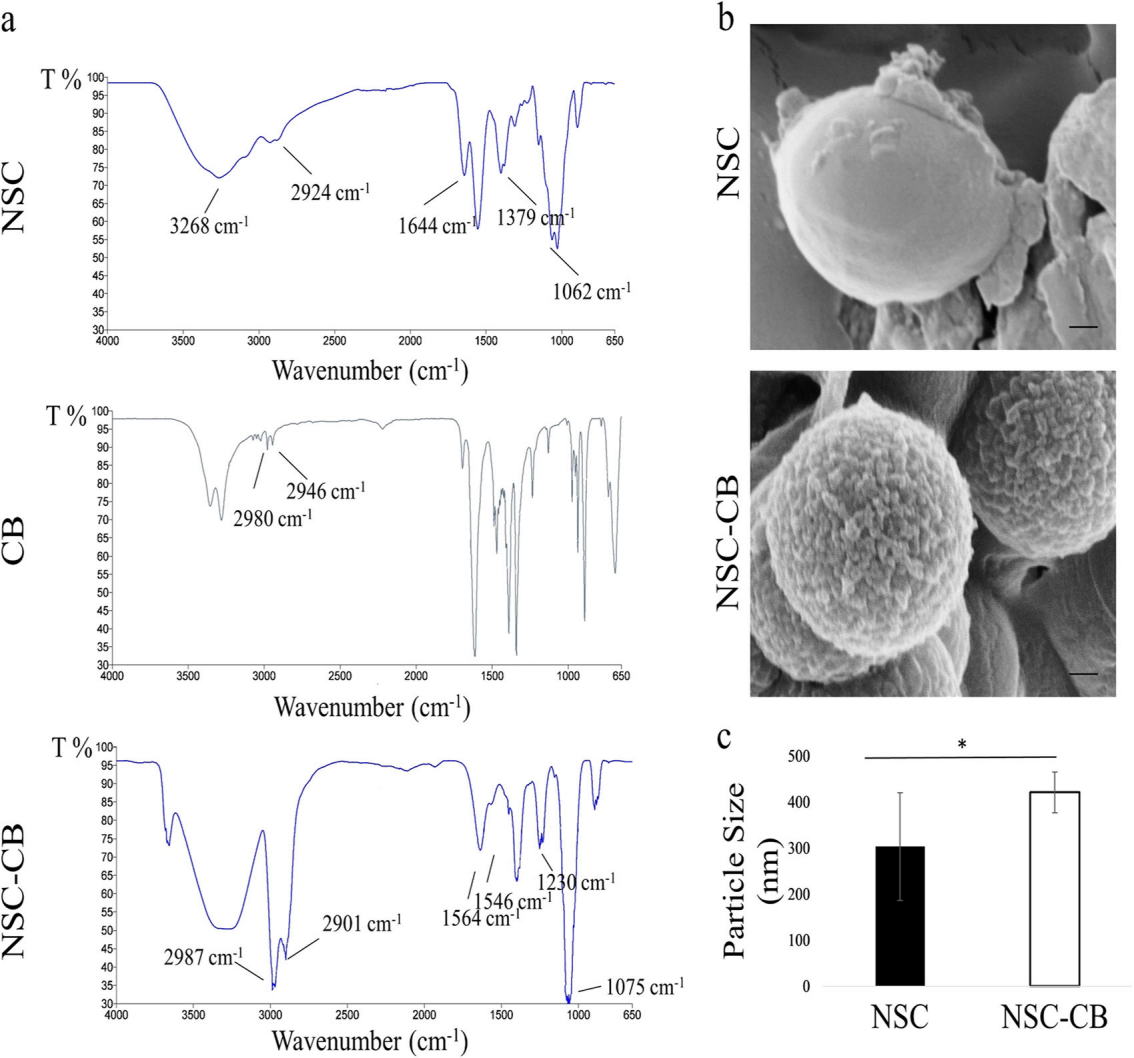
Characterisation of NSC with and without CB. **a** FTIR; **b** SEM (scale bar is 1 µm) and **c** DLS analysis

The successful succinylation of the chitosan was confirmed by FTIR (Fig. 1a). In NSC absorption bands were visible at 2924 cm^−1^ (stretching of –CH_2_-), 3268 and 1062 cm^−1^ (amino group characteristics), 1644 cm^−1^ (Amide I) and 1379 cm^−1^ (Amide III) consistent with previous studies [30]. Compared to the NSC spectrum, NSC-CB showed distinct absorption bands attributed to the reduction of the peaks at 1564 and 1546 cm^−1^ (primary amine groups), increase of peaks at 2901 and 2987 cm^−1^, observed only in the CB spectrum, and presence of peaks between 1075 and 1230 cm^−1^ related to the aliphatic amine region [31]. Such successful modification was supported by changes in structure and surface morphology as shown in Fig. 1b. NSC appeared almost spherical shape with a smooth surface that appeared rough once modified with CB. NSC-CB were seen to be slightly bigger in size (421 ± 44 nm) when compared with NSC (303 nm ± 117 nm) (Fig. 1c).

### NSC-CB encourage MIN6 cells to spontaneously aggregate and form spheroids

**Fig. 2:**
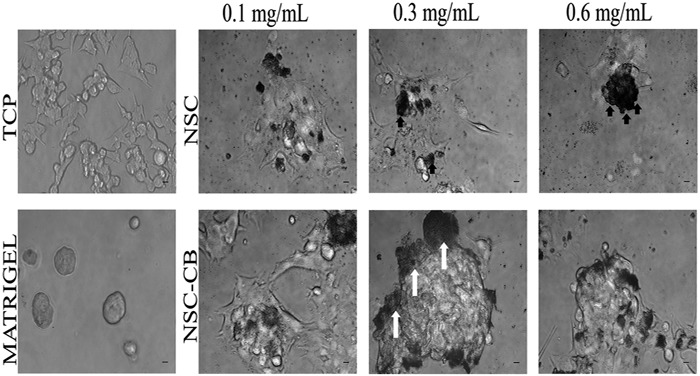
Morphological changes of MIN6 cells cultured in various NSC conditions after 48 h incubation. White arrows indicate the formation of aggregates made by NSC-CB at 0.3 mg/mL. Scale bar is 100 µm

At 48 h incubation, MIN6 cells grown onto TCP spread and appeared of regular shape compared to those treated with NSC at all concentrations tested (Fig. 2). NSC-treated cells failed to reach a completely confluent state especially those with NSC at 0.6 mg/mL concentrations which have been observed to be entrapped by close-packed beads. In contrast, cells treated with NSC-CB at 0.1 mg/mL became organised into larger monolayers that spontaneously aggregated together to form 3D spheroids when in contact with higher NSC-CB concentrations as observed in Matrigel-treated MIN6 cells. However, 0.3 mg/mL NSC-CB gave rise to larger and more compact spheroids compared to 0.6 mg/mL concentrations where presence of monolayer MIN6 cells were still visible. At 48 h, 0.3 mg/mL NSC-CB formed larger aggregates that were predominantly restricted to the periphery of spheroids (white arrows).

**Fig. 3:**
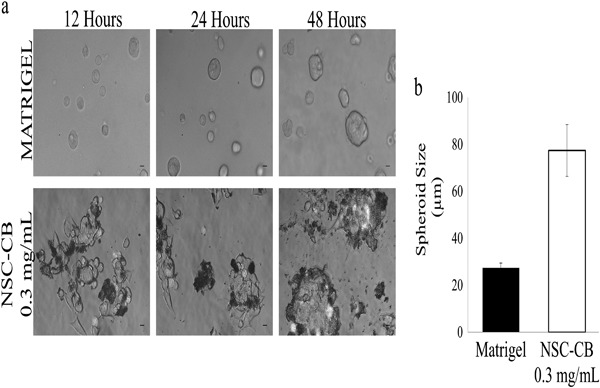
Spheroid formation. **a** Phase contrast images (scale bar is 100 µm) and **b** size of spheroids formed onto Matrigel substrates and NSC-CB at 0.3 mg/mL. *P ≤ 0.01; mean ± SD; *n* = 12

As suggested by the micrographs, Matrigel substrates maintained MIN6 cell clusters in their spheroidal geometry during a 12–48-h culture period; whereas NSC-CB at 0.3 mg/mL concentrations resulted in individual cells spontaneously assembling in tight and larger spheroids (Fig. 3a). The size of these spheroids was found to be larger [77.03 ± 11.08 µm] than those cultured onto Matrigel substrates [27.41 ± 2.07 µm] as shown in Fig. 3b.

### NSC-CB reduce cytotoxic effects of NSC and improve MIN6 cell proliferation

**Fig. 4:**
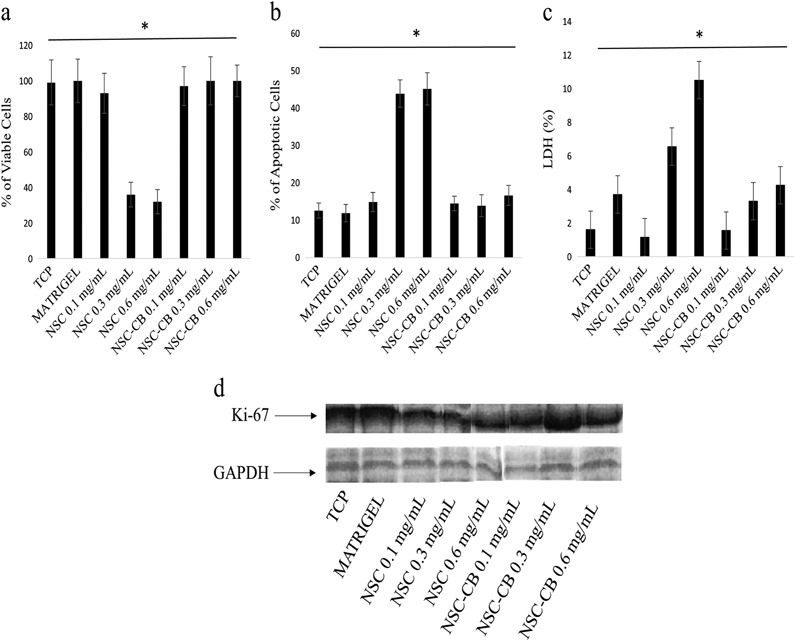
Detection of toxicity and cell proliferation of MIN6 cells treated with NSC and NSC-CB at distinct concentrations after 48 h incubation. Percentage of **a** viable cells, **b** apoptosis and **c** LDH (* *P* ≤ 0.05, mean ± SD compared TCP; *n* = 12); **d** western blotting analysis of Ki-67 expression compared to a positive control, GAPDH

The measurement of HPI staining demonstrated that cell viability was higher, with more than 99% within all tested cells. However, these levels were lower in those treated with NSC at 0.3 and 0.6 mg/mL concentrations (~30%) (Fig. 4a). At these concentrations, a twofold increase in the percentage of apoptotic cells was also found (~50%) when compared with control substrates and NSC-CB at 0.1, 0.3 and 0.6 mg/mL (~10%) (Fig. 4b). These results were corroborated by LDH data where elevated levels of LDH were released by NSC-treated monolayer cells at 0.3 and 0.6 mg/mL when compared to NSC-CB of comparative concentrations and within the range of the control cells at 48 h (Fig. 4c). However, MIN6 cells treated with both 0.3 and 0.6 mg/mL NSC-CB released slightly higher amounts of LDH (~4%) than cells onto TCP (~2%) which was linked to anoikis [30] rather than material toxicity as similar effects were detected within MIN6 spheroids seeded onto Matrigel substrates (~4%). The presence of dead cells at the centre of the NSC-CB-treated spheroids at 0.3 and 0.6 mg/mL was also revealed in Matrigel-forming spheroids (Supplementary Data Fig. 1). Interestingly, Ki-67 protein expression, a marker for proliferation, demonstrated that MIN6 cells grew at a higher rate when treated with NSC-CB and control substrates rather than NSC at 0.1, 0.3 and 0.6 mg/mL after 48 h of incubation (Fig. 4d).

### E-cadherin regulation is upregulated within MIN6 cells by NSC-CB

**Fig. 5:**
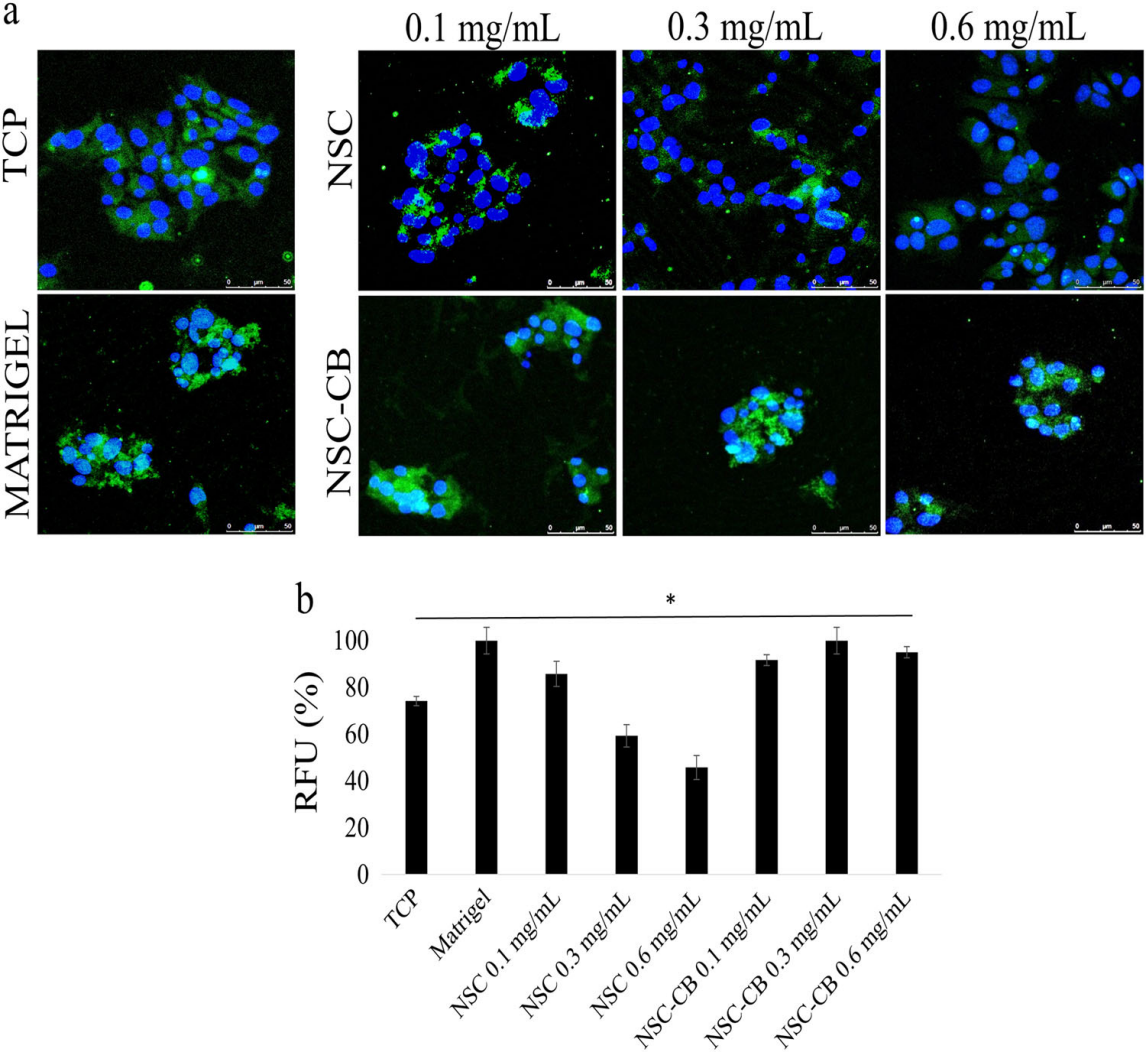
Functional expression of E-cadherin activated within monolayer and spheroid-forming MIN6 cells tested with non-modified and modified NSC. **a** Localisation of E-cadherin is shown in green while blue represents DAPI staining of the nucleus (scale bar is 50 µm); **b** E-cadherin expression was quantified by measuring relative fluorescence unit (RFU) of the immunostaining. *P ≤ 0.01, mean ± SD; *n* = 12 (color figure online)

An enhanced presence of cell–cell junctions was demonstrated by a greater expression of the intercellular binding protein E-cadherin within MIN6 cells treated with NSC-CB compared with those with NSC at higher concentrations (Fig. 5a). This was evenly distributed throughout the cytoplasm of both individual MIN6 cells and spheroids, but sharply declined to undetectable levels (CTCF was 1.5- to 2-fold decrease) within NSC-treating cells at 0.3 and 0.6 mg/mL, respectively (Fig. 5b).

### NSC-CB-treated cell spheroids store and release insulin while highly express Pdx-1

**Fig. 6:**
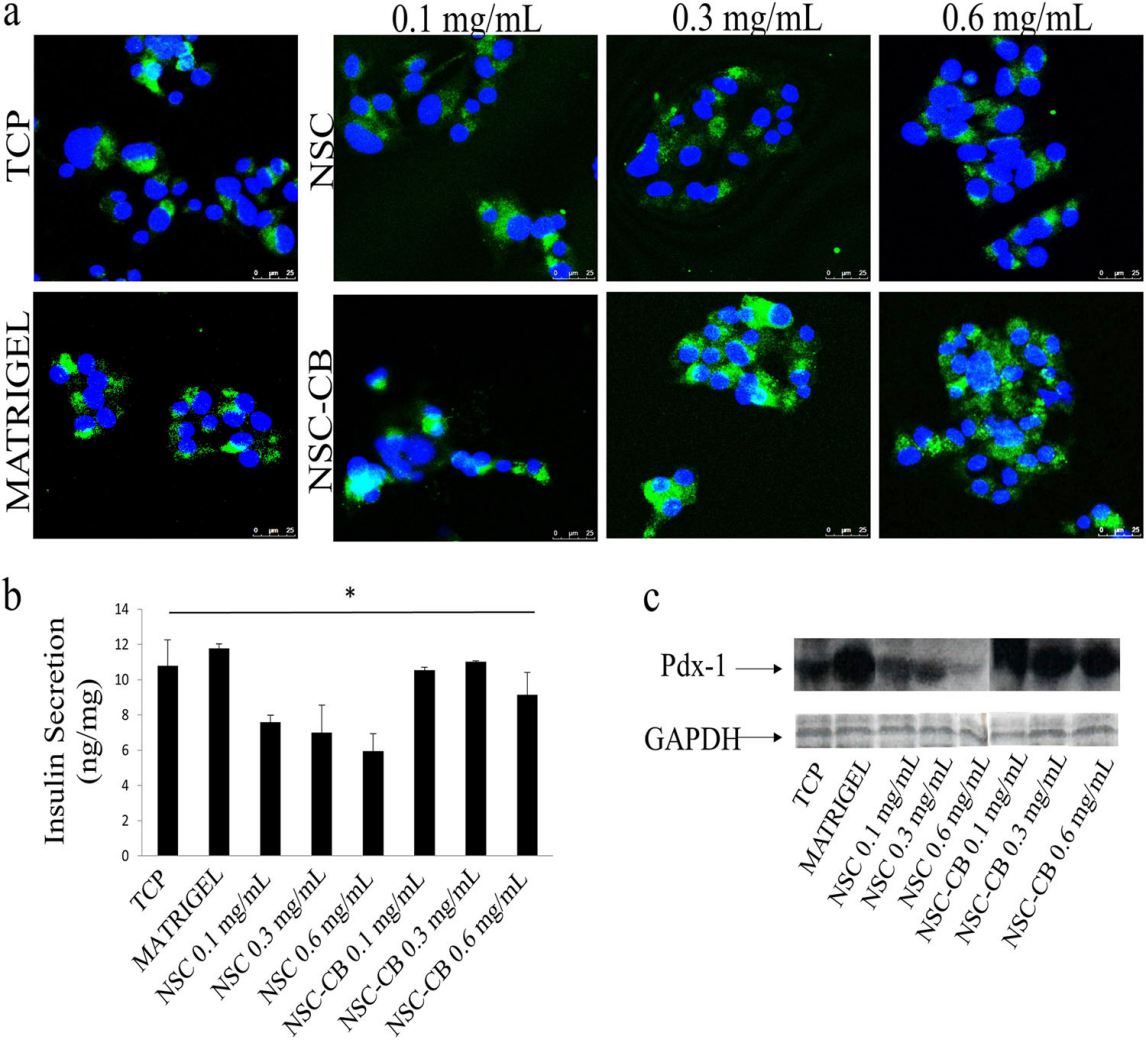
Regulation of both insulin and Pdx-1 proteins in MIN6 cells seeded with NSC and NSC-CB at 0.1, 0.3 and 0.6 mg/mL. **a** Insulin storage and **b** release by cells at 48 h (scale bar is 25 µm; *P ≤ 0.01; mean ± SD; *n* = 6); **c** characterisation of Pdx-1 expression analysed related to GAPDH by Western blotting

To test whether NSC with and without CB were able to maintain insulin expression throughout aggregation and spheroid formation, MIN6 cells were assessed for their ability to store (Fig. 6a) and release insulin (Fig. 6b). It was found that all MIN6 cells were positive for insulin content, particularly those seeded with NSC-CB. A stronger signal was detected in the cytoplasm of each spheroid after 48 h in vitro culture. However, the levels of insulin secreted by NSC-CB-treated MIN6 cells [0.1 mg/mL = 10.5 ± 1.2 ng/mg; 0.3 mg/mL = 11.01 ± 0.02 ng/mg; 0.6 mg/mL = 9.14 ± 1.5 ng/mg] were found to be similar of those of TCP [10.7 ± 1.3 ng/mg] and Matrigel control [11.7 ± 1.2 ng/mg]; whereas NSC-treated cells demonstrated impaired insulin secretion [0.1 mg/mL = 7.5 ± 1.3 ng/mg; 0.3 mg/mL = 7 ± 0.79 ng/mg; 0.6 mg/mL = 5.9 ± 1.08 ng/mg]. Western blot analysis also displayed increased protein expression levels of Pdx-1 within both control cells and those grown with NSC-CB compared to NSC at all concentrations tested as shown in Fig. 6c.

## Discussion

The lack of truly representative in vitro models of the pancreatic islet hinder the elucidation of the mechanisms involved in the maintenance of their viability and function. Pancreatic β-cells in particular are generally considered to be a difficult cell type to keep alive and active in vitro [32]. Within 48–72 h of culture, the cells usually lose their ability to secrete insulin and to express critical transcription factors (e.g. Pdx-1) [33]. Recent studies have demonstrated that both cell–cell and cell–matrix interactions are key regulators with the ability to induce or repress β-cell identity and function and as such represent key features of the pancreatic microenvironment [34]. In light of this, 3D cellular spheroid systems have attracted much attention as the increased cell-to-cell interactions in spheroids have been demonstrated to enhance cell survival and functioning after transplantation into the body [8, 35]. A number of different approaches have been developed for the aggregation of cells into spheroids [36], but all still face distinct disadvantages including batch-to-batch variation, being relatively labour-intensive and prohibitively costly (e.g. Matrigel).

This study has taken a novel, cost effective approach to encourage the aggregation of β-cells using CB-functionalised succinylated chitosan beads to form functional β-cell spheroids using conventional 2D tissue culture techniques. The rationale for this approach was driven by the potential for the zwitterionic coating to allow interaction with the cell membrane glycocalyx that helps cells to bind with each other and to retain water while providing intercellular adherences and elaboration of specific chemical cues [36]. The MIN6 insulinoma cell line was used in this study to demonstrate that NSC-CB had the ability to promote both cellular recruitment and the spontaneous formation of spheroids within 48 h of culture, negating the use of complex equipment employed in previous attempts to produce pseudoislets in vitro [9]. Thus, the successful grafting of CB onto the surface of NSC via simple carbodiimide chemistry under mild conditions was shown to provide an ideal matrix for the loading of these zwitterionic units to supply carboxyl groups enabling bio-conjugation. However, the zwitterionic properties of CB are known to be lost during the crosslinking process that allows CB to be grafted onto NSC surfaces [37]. Nonetheless, when re-suspended with MIN6 cells in medium, NSC-CB at a concentration of 0.3 mg/mL encouraged the formation of cellular spheroids that increased in size within 48 h culture. It is proposed that following the initial rapid preparation of NSC-CB, the decrease in intermolecular and intramolecular hydrogen bonding becomes weaker, resulting in increased self-aggregation of NSC-CB once mixed with the cells in medium. Within 48 h of culture, these beads aggregated to form larger constructs that tightly influenced the morphology, viability and activities of the MIN6 cells. However, at higher concentrations of the functionalised beads-treated MIN6 cells were induced to become immature while showing an irreversibly inhibited proliferation, possible due to a decrease in net negative surface charges on the MIN6 cell membrane as previously reported [38, 39]; this in turn likely led MIN6 cells to be more susceptible to apoptosis and hence death. When grafted onto NSC surfaces, CB not only improved cell survival, but also showed the capability to stimulate cell-to-cell interactions at the same levels as those observed when TCP and Matrigel substrates were used.

However, only 0.3 mg/mL SCN-CB generated spheroids with dimensions similar to those seen in a native islet (~80 µm) [40, 41]. Although the size of these spheroids was variable, unlike those observed on Matrigel substrates, it can be suggested that they resemble more closely the distribution seen in vivo where β-cells migrate from islets of distinct sizes, probably as a way of maximising their exposure to blood vessels [42].

Moreover, 0.3 mg/mL NSC-CB-forming spheroids formed and preserved higher levels of intracellular insulin contents as an indicator of a physiologically-like β-cell function after up to 48 h in culture. Although, their insulin secretory response was found to be slightly higher to that within MIN6 cells grown onto control substrates, these levels of insulin were predicted and are known to be related to the cell viability data. The lack of oxygen severely influenced insulin release from cells confined to the centre of spheroids which are known to be more prone to hypoxia and indeed apoptosis [43]. It was also notable that NSC-CB-forming spheroids grew at the same rate as those cultured onto Matrigel substrates, presumably as a result of the enhanced cell-to cell and cell–matrix contacts.

The precise correlation between E-cadherin and insulin is still uncertain, but highly E-cadherin expressing NSC-CB treated MIN6 cell spheroids were shown in this study to secrete more insulin than those cultured with NSC at equivalent concentrations. The strongest expression of E-cadherin was observed in the more central part of SNC-CB-treated MIN6 cell spheroids corresponding to cells that showed the highest levels of insulin activity. However, downregulation of E-cadherin negatively affected insulin secretion within monolayers cultured with higher concentrations of NSC suggesting that the loss of E-cadherin led cells to be functionally inactive. Recent studies have suggested a relationship between E-cadherin and insulin production as a function of cell proliferation [44], which may explain the results seen in this study. It appears that the functional connection between cell growth and this signalling pathway likely involves Pdx-1, a regulator of β-cell differentiation and survival [45]. Evidence has been reported to suggest that reduced Pdx-1 expression induces impaired insulin secretion which is commonly associated with abnormal β-cell morphology and decreased cell growth and differentiation [46]. In accordance with this, the amounts of both Ki-67 and Pdx-1 differed within non-mature MIN6 cells treated with 0.3 and 0.6 mg/mL NSC, corresponding to their lower insulin activity. Hence, the selective modification of NSC with CB, both chemically and topographically, appeared to be essential for the normal cellular development and function of MIN6 cells. By controlling the delicate balance between cell apoptosis and maturation, 0.3 mg/mL NSC-CB drastically promoted proliferation of MIN6 cells within spheroids by enhancing their cell-to-cell interactions with potential repercussions for insulin secretion.

In conclusion, NSC-CB has proven to be a valuable method for the rapid and spontaneous generation of functional β-cell spheroids. Among a wide range of cell culture systems, it provides a simple, fast, reproducible and cost-effective tool for the production of viable and functional spheroids resembling the in vivo features of a cellular microenvironment. This novel approach provides a new model for in vitro studies aiming to elucidate the cellular and molecular dynamics involved in the initiation and progression of diabetes and preliminary testing of new therapeutics.

## Electronic supplementary material

Supplementary Data Fig. 1 Comparison of apoptotic effects within MIN6 spheroids grown onto Matrigel substrates and NSC-CB at 0.3 mg/mL. Viable cells are shown as blue dots while apoptotic cells are in pink staining. Scale bar is 100 µm
