# Supplementary figures and images for: Carboxybetaine-modified succinylated chitosan-based beads encourage pancreatic β-cells (Min-6) to form islet-like spheroids under in vitro conditions
Source: J Mater Sci Mater Med. 2017 Dec 30;29(1):15. doi: 10.1007/s10856-017-6018-0 (PMC5748029; doi:10.1007/s10856-017-6018-0)

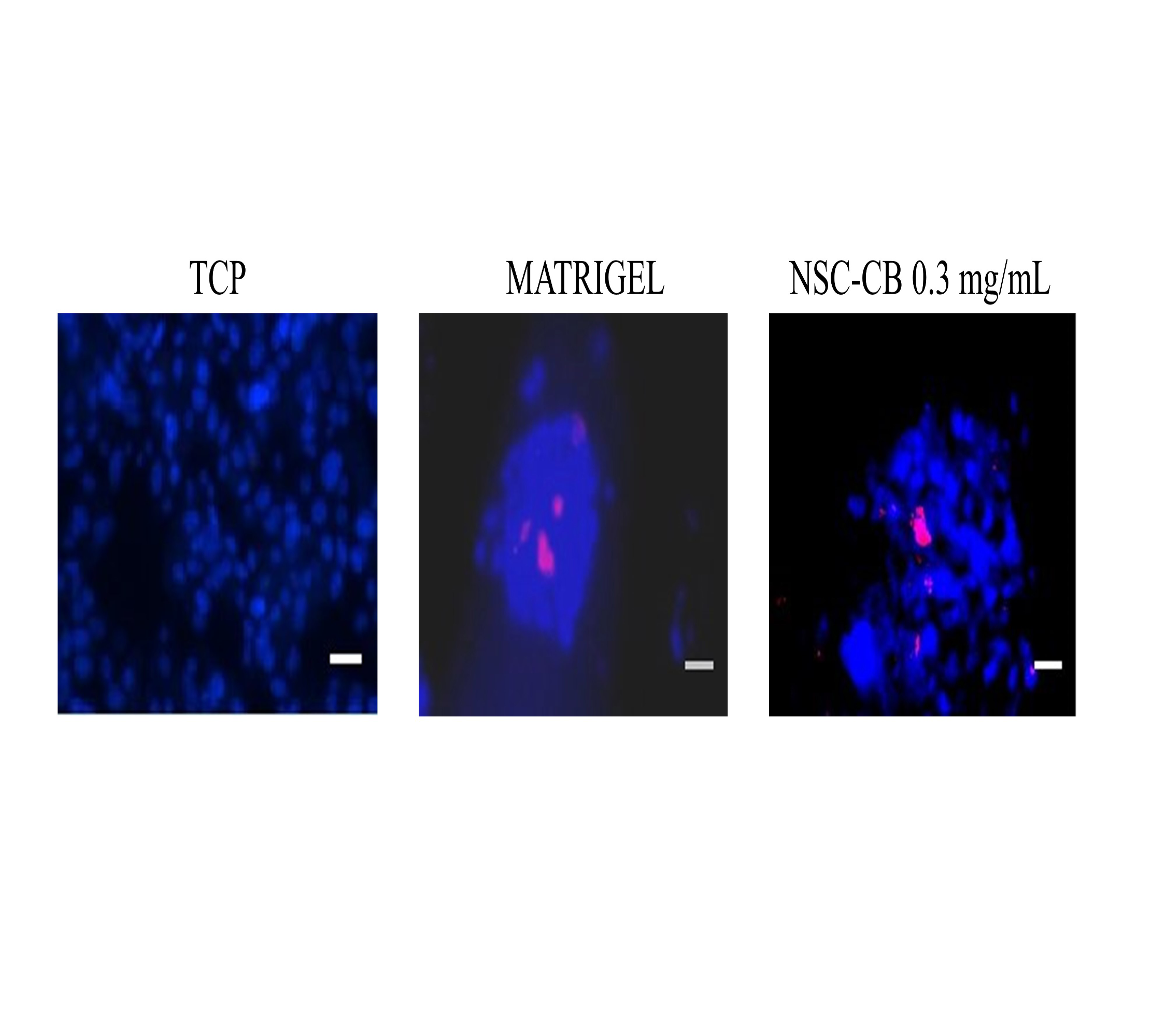

Supplement: Supplementary file 1 — Supplementary Data Fig. 1 Comparison of apoptotic effects within MIN6 spheroids grown onto Matrigel substrates and NSC-CB at 0.3 mg/mL. Viable cells are shown as blue dots while apoptotic cells are in pink staining. Scale bar is 100 µm [file 10856_2017_6018_MOESM1_ESM.1]
